# Impressive extramedullary plasmacytoma response in refractory multiple myeloma treated with teclistamab

**DOI:** 10.1002/jha2.821

**Published:** 2023-11-23

**Authors:** Intissar Ben Salah, Lydiane Mordier, Charlotte Leleux, Clément Gourguechon, Delphine Lebon, Lydia Montes

**Affiliations:** ^1^ Haematology Department CHU Amiens Amiens France; ^2^ Internal Medicine CHU Amiens Amiens France

1

A 70‐year‐old patient was addressed in October 2010 as part of an etiological work‐up for chronic sinusitis, for hyperproteinemia at 147 g/l (normal 57–82 g/l) with a peak at 68.8 g/l on serum IgG lambda protein electrophoresis. Multiple myeloma was diagnosed on bone marrow aspiration with translocation t(4;14) on cytogenetics analysis, which confers intermediate prognosis if no additional cytogenetic abnormalities. From 2010 to 2022, the patient received multiple lines of treatment including proteasome inhibitors (bortezomib, ixazomib and carfilzomib), immunomodulators (thalidomide, lenalidomide and pomalidomide), monoclonal antibodies (daratumumab, belantamab mafodotin), chemotherapies (doxorubicin, cyclophosphamide, etoposide and melphalan) and autologous stem‐cell transplantation.

He relapsed in September 2022 and experienced a fast and tumoral progression with cutaneous and sub‐cutaneous plasmacytomas, located on the right deltoid and supraclavicular regions (Figure 1). We supposed that he presented a liver plasmacytoma also but we did not biopsy, described on CT scan. In October 2022, teclistamab treatment was started (BCMA chimeric antigen receptor‐T cells were not available). Teclistamab is a T‐cell–redirecting bispecific antibody that targets both CD3 expressed on the surface of T cells and B‐cell maturation antigen expressed on the surface of plasma cells involved in multiple myeloma proliferation. In monotherapy, over patients with past several treatments, including triple‐class refractory, teclistamab achieved an overall response of 63% and median duration of response is 18.4 months. Our patient attended a grade 1 cytokine release syndrome during ramp up phase treated with supportive care and lasted 24 hours. Partial response was assessed one month after treatment initiation with complete disappearance of skin lesions and >50% reduction peak level (Figure 2). Best response experienced was VGPR after 3 months with M‐protein levels undetectable, no skin lesions and liver tumoral reduction at CT scan. He suffered from hypogammaglobulinemia and needed immunoglobulin infusions to avoid infectious adverse events. Unfortunately, our patient relapsed with new bone osteolytic lesions at 7 months.

**FIGURE 1:**
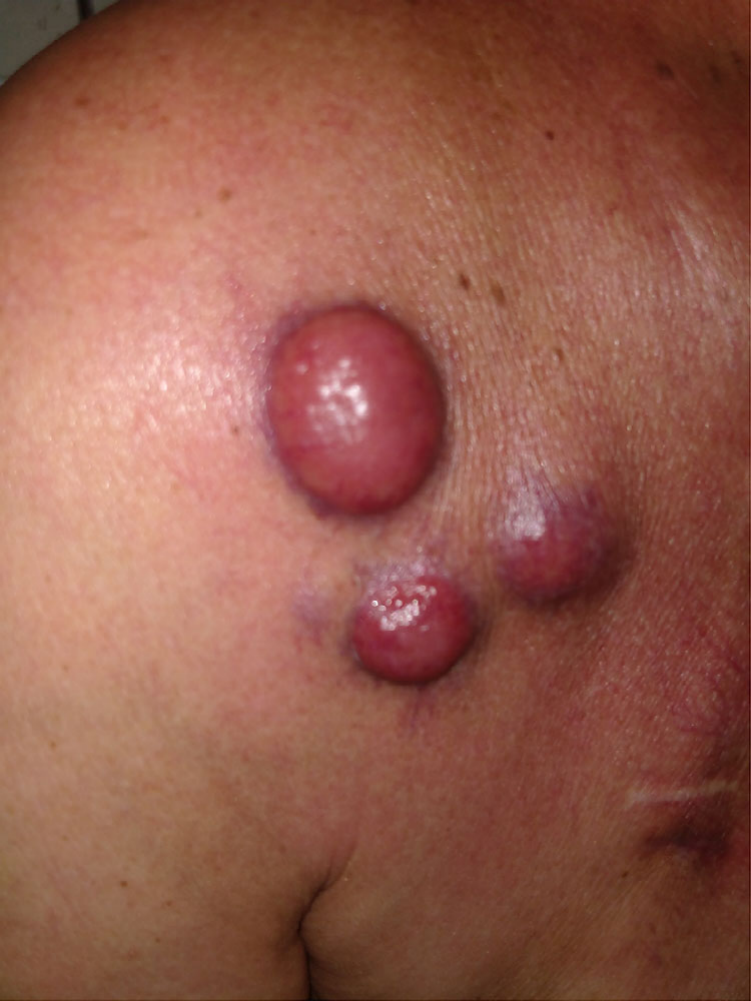
Cutaneous plasmacytomas on right deltoid and supra‐clavicular region, at relapse.

**FIGURE 2:**
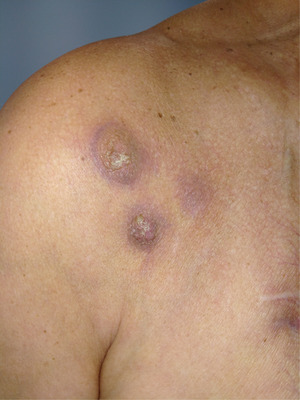
Cutaneous plasmacytomas after teclistamab treatment.

This case highlights fast and impressive response in a refractory multiple myeloma patient with extramedullary disease treated with Teclistamab, a BCMA bispecific antibody. This is an interesting report, particularly because clinical trials with bispecific reported poor response among patients with extramedullary disease. Moreover, our patient was treated with belantamab mafodotin, a BCMA drug conjugate monoclonal antibody, which could cause a BCMA target loss and so a less good response to BCMA bispecific.

## AUTHOR CONTRIBUTIONS

IB and LM wrote the manuscript. LM, CL, CG, DL and LM revised the manuscript.

## CONFLICT OF INTEREST STATEMENT

The authors declare no conflict of interest.

## FUNDING INFORMATION

The authors received no specific funding for this work.

## ETHICS STATEMENT

Local ethics approval statement was achieved.

## PATIENT CONSENT STATEMENT

Patient consent statement was achieved.

## Data Availability

Data sharing is not applicable to this article as no new data were created or analyzed in this study.

